# A Novel CDK4/6 and PARP Dual Inhibitor ZC-22 Effectively Suppresses Tumor Growth and Improves the Response to Cisplatin Treatment in Breast and Ovarian Cancer

**DOI:** 10.3390/ijms23052892

**Published:** 2022-03-07

**Authors:** Chenchen Tian, Yufan Wei, Jianjun Li, Zhi Huang, Qiong Wang, Yingxue Lin, Xingping Lv, Yanan Chen, Yan Fan, Peiqing Sun, Rong Xiang, Antao Chang, Shuang Yang

**Affiliations:** 1 Tianjin Key Laboratory of Tumor Microenvironment and Neurovascular Regulation, School of Medicine, Nankai University, Tianjin 300071, China; tianchenchen@ucas.ac.cn (C.T.); weiyufan@mail.nankai.edu.cn (Y.W.); lijianjun@mail.nankai.edu.cn (J.L.); huangzhi@mail.nankai.edu.cn (Z.H.); qiongwang2022@gmail.com (Q.W.); linyingxue@mail.nankai.edu.cn (Y.L.); lvxingping107@mail.nankai.edu.cn (X.L.); chenyanan@nankai.edu.cn (Y.C.); yanfan@nankai.edu.cn (Y.F.); rxiang@nankai.edu.cn (R.X.); 2 Department of Cancer Biology, Comprehensive Cancer Center, Wake Forest Baptist Medical Center, Winston Salem, NC 27157, USA; psun@wakehealth.edu; 3 Department of Pancreatic Cancer, Tianjin Medical University Cancer Institute and Hospital, Tianjin 300060, China

**Keywords:** breast cancer, ovarian cancer, ZC-22, PARP inhibitor, CDK4/6 inhibitor

## Abstract

In recent years, three PARP inhibitors and three CDK4/6 inhibitors have been approved by the FDA for the treatment of recurrent ovarian cancer and advanced ER-positive breast cancer, respectively. However, the clinical benefits of the PARPi or CDK4/6i monotherapy are not as satisfied as expected and benefit only a fraction of patients. Current studies have shown therapeutic synergy for combinations of PARPi and CDK4/6i in breast and ovarian cancers with homologous recombination (HR) proficiency, which represents a new synthetic lethal strategy for treatment of these cancers regardless HR status. Thus, any compounds or strategies that can combine PARP and CDK4/6 inhibition will likely have great potential in improving clinic outcomes and in benefiting more patients. In this study, we developed a novel compound, ZC-22, that effectively inhibited both PARP and CDK4/6. This dual-targeting compound significantly inhibited breast and ovarian cancer cells by inducing cell cycle arrest and severe DNA damage both in vitro and in vivo. Interestingly, the efficacy of ZC-22 is even higher than the combination of PARPi Olaparib and CDK4/6i Abemaciclib in most breast and ovarian cancer cells, suggesting that it may be an effective alternative for the PARPi and CDK4/6i combination therapy. Moreover, ZC-22 sensitized breast and ovarian cancer cells to cisplatin treatment, a widely used chemotherapeutic agent. Altogether, our study has demonstrated the potency of a novel CDK4/6 and PARP dual inhibitor, which can potentially be developed into a monotherapy or combinatorial therapy with cisplatin for breast and ovarian cancer patients with HR proficiency.

## 1. Introduction

Breast cancer is the leading cause of cancer morbidity and mortality in women worldwide, accounting for 24.2% of new cancer cases and 15.0% of cancer deaths [1]. Ovarian cancer, although not as frequent, is the most fatal of all female reproductive cancers, representing 4.4% of cancer deaths worldwide [1,2]. Currently, several types of targeted drugs, such as cyclin-dependent kinase 4/6 (CDK4/6) inhibitors (CDK4/6i), poly-(ADP)-ribose polymerase (PARP) inhibitors (PARPi), PI3K inhibitors, and AKT inhibitors, are approved for treating certain subtypes of breast cancer and ovarian cancer, but none of these drugs alone showed satisfactory clinical benefits [3,4], suggesting an urgent need and immediate significance for discovery of new biomarkers of responses, and new strategies of combinatorial treatments.

CDK4/6 play pivotal roles in the transition from the G1 to S phases by regulating the phosphorylation state of retinoblastoma-associated protein (Rb) and thus are essential for cell-cycle progression in many cancer cells. Due to the importance of CDK4/6 activity in cancer cells, CDK4/6 inhibitors have emerged as promising candidates for cancer treatments [5,6]. To date, three highly selective CDK4/6i, Palbociclib (PD0332991), Ribociclib (LEE0011), and Abemaciclib (LY2835219), have been approved by FDA as first-line therapy for the treatment of hormone receptor-positive (HR^+^) and human epidermal growth factor receptor 2-negative (HER2^−^) metastatic breast cancer in combination with endocrine therapy [7,8,9,10]. Recently, CDK4/6i have shown potent antitumor activity in ovarian cancer either as single agent or in combination with platinum-based chemotherapy in several clinical trials [9,11,12]. However, the clinical benefits of CDK4/6i are limited and were only observed in a fraction of breast and ovarian cancer patients [12,13]. The combination of CDK4/6 inhibitors with other agents might be a more effective strategy to improve clinical outcomes and to extend the use of CDK4/6 inhibitors to a broader spectrum of breast and ovarian cancer patients than CDK4/6i alone.

PARP1/2 are post-translational modification enzymes that are pivotal in the repair of single-strand DNA breaks (SSBs) by the base-excision repair (BER) pathway in normal and cancer cells [14,15]. Inhibition of PARP1/2 leads to the accumulation of single-strand breaks and, consequently, double-stranded DNA breaks (DSBs), which must be repaired by the homologous recombination (HR) or non-homologous end-joining (NHEJ) pathways for cell survival [16]. Thus far, three PARP inhibitors, Olaparib, Rucaparib, and Niraparib, have been approved by FDA for treatment of breast and ovarian cancers in patients with mutations in BRCA1 and BRCA2, which are key mediators of HR repair, providing the first anti-cancer therapy based on synthetic lethality [16,17,18]. However, the efficacy of PARPi in the treatment of breast and ovarian cancers is mitigated by the fact that most cancers with intrinsic HR proficiency do not respond as well as HR-deficient cancers [19,20]. Therapeutically, combinations of PARPi with other targeted drugs that inhibit HR might be an effective approach to expand their use beyond HR-deficient cancers [16,21].

Recently, therapeutic synergy for combination of PARPi and CDK4/6i has been demonstrated in MYC highly expressed breast and ovarian cancers with HR proficiency [22,23], which provides a new synthetic lethal strategy for treatment of these cancers regardless HR status. Thus, any strategies or compounds that could combine PARP and CDK4/6 inhibition may improve the clinic outcomes and benefit more patients with breast or ovarian cancer. In this study, we developed a new compound ZC-22, which can effectively inhibit the activity of both PARP and CDK4/6 and displayed better anti-tumor efficacy than PARPi Olaparib and CDK4/6i Abemaciclib monotherapy, or even combination therapy in both cell and mouse models. In addition, ZC-22 also greatly improved the response of breast and ovarian cancer cells to cisplatin treatment, which is a widely used chemotherapeutic agent. We have thus developed a novel compound that can potentially benefit breast and ovarian cancer patients with primary or secondary HR proficiency as monotherapy or in combination with platinum-based chemotherapy.

## 2. Materials and Methods

### 2.1. Cell Culture

Human breast cancer cell lines MDA-MB-231 and SUM-159, and human ovarian cancer cell lines OVCAR5 and SKOV3 were obtained from the American Type Culture Collection (ATCC). MDA-MB-231 cells were cultured in L-15 medium with 10% fetal bovine serum (FBS), incubated at 37 °C in a humidified incubator without additional CO_2_. SUM-159 cells were maintained in Dulbecco’s modified Eagle’s medium (DMEM) containing 10% FBS, and OVCAR5 and SKOV3 cells were incubated in Roswell Park Memorial Institute (RPMI)-1640 medium with 10% FBS and cultured at 37 °C in a humidified incubator containing 5% CO_2_.

### 2.2. Antibodies and Reagents

CDK4/6i Abemaciclib (LY2835219) was purchased from Eli Lily and Company (Indianapolis, IN, USA), PARPi Olaparib was from Astrazeneca (Wilmington, DE, USA), and cisplatin was from MedChemExpress (Monmouth Junction, NJ, USA). The anti-CDK4 (#12790), anti-CDK6 (#13331), anti-Rb (#9309), anti-p-Rb (#8516), anti-γH2A.X (#9718), and anti-caspase3 (#9662) antibodies were purchased from Cell Signaling Technology (Danvers, MA, USA). The anti-β-actin (sc-47778) antibody was purchased from Santa Cruz Biotechnology (Dallas, TX, USA), anti-Ki67 (ab16667) antibody was from Abcam (Cambridge, UK), anti-H2A.X (10856-1-AP) antibody was from Proteintech (Chicago, IL, USA), anti-PARP (SRP00944) antibody was from Saierbio (Tianjin, China), and anti-PAR (4336-BPC-100) antibody was from Trevigen (Gaithersburg, MD, USA).

### 2.3. Cell Viability Assay

Cell viability assay was performed as described before [24]. Briefly, 2500/well of breast or ovarian cancer cells were seeded into a 96-well plate; grown overnight; treated with indicated concentration of Olaparib, LY2835219, ZC22, LP-1, GC-24, or cisplatin alone or together for an indicated amount of time; and then incubated with Cell Counting kit-8 (CCK-8) substrate (Dojindo, Kumamoto, Kyushu Island, Japan) for 1–2 h at 37 °C. Cell viability was calculated by the absorbance at 450 nm measured by the GloMax Explorer (Promega, Madison, WI, USA). Cells without treatment were set as 100%, and medium control (no cells) was set as 0%.

### 2.4. Western Blot Analysis

Cells were washed with PBS, collected with scraper, and then lysed in RIPA buffer containing 1× protease and phosphatase inhibitors for 30 min on ice. The supernatants were collected by centrifugation at full speed for 10 min at 4 °C and subjected to SDS-PAGE followed by immunoblotting as described before [25]. Signals were detected using enhanced chemiluminescence (Millipore, Burlington, MA, USA) and captured by the G: BOX Chemi XRQ gel doc System (Syngene, Cambridge, UK).

### 2.5. Cell Cycle Analysis

MDA-MB-231 cells were treated with 2 μM LY2835219 or ZC-22, and OVCAR5 cells were treated with 0.5 μM LY2835219 or ZC-22 for 24 h. After washing in PBS, cells were fixed with 70% ethanol on ice for 2 h, followed by centrifugation at 300× *g*—for 20 min at 4 °C, and then subjected to a propidium iodide (PI, BD Biosciences, Franklin Lakes, NJ, USA) based cell-cycle analysis according to the manufacturer’s protocols. Cell-cycle distribution was analyzed by flow cytometry. Each experiment was performed in triplicates.

### 2.6. Cell Apoptosis Analysis

MDA-MB-231 and OVCAR5 cells were treated with indicated concentration of LY2835219 or ZC-22 for 48 h. The cells, together with the supernatants, were collected and subjected to apoptosis analysis using the FITC Annexin-V/PI apoptosis detection kit (BD Biosciences), following the manufacturer’s protocols. Percentage of apoptotic cells was assessed by flow cytometry. Each experiment was performed in triplicates.

### 2.7. EdU Incorporation Assay

The indicated concentration of cells were seeded on cover glasses in 24-well plates; cultured for 24 h; and then treated with cisplatin alone or together with LY2835219, Olaparib, or ZC-22 for 24 h, followed by incubation with 10 µM EdU for 2 h at 37 °C. After washing in PBS, cells were stained with the EdU assay kit (iFluor 488, Abcam) according to manufacturer’s protocols. EdU incorporating cells were analyzed by a fluoresce microscope. About 100 cells/field and a total of three fields were counted. Each experiment was performed in triplicates.

### 2.8. Immunofluorescence

Immunofluorescence-staining of γH2A.X was performed as described before [24]. Briefly, cells were seeded on cover glasses in 24-well plates, cultured for 24–48 h, and then treated with cisplatin alone or together with Olaparib or ZC-22 for 24 h. After washing with PBS, cells were fixed by 4% PFA, permeated with 0.5% Triton-X100 in PBS, washed thrice with 0.1% PBS-Tween (PBST), blocked with 3% BSA in PBST, and then incubated with anti-γH2A.X antibody overnight at 4 °C. Cells were then washed thrice with 0.1% PBST, incubated with Alexa Fluor 488 conjugated secondary antibody (Cell Signaling, Danvers, MA, USA) for 1 h at room temperature in dark, and mounted with Antifade Mounting Medium with DAPI (Vector Laboratories, Burlingame, CA, USA). Images were obtained with the Olympus FV1000 laser scanning confocal microscope. At least 150 cells in three fields were counted for each repeat.

### 2.9. Xenograft Mice Models

Six-week-old female athymic nude mice (Homozygous) were purchase from Vital River Laboratories (Beijing, China) and maintained under the guidelines for laboratory animals of Nankai university (NKU). Mice were injected subcutaneously with 2 × 10^6^ MDA-MB-231 or 8 × 10^6^ OVCAR5 cells. When tumors were 50–100 mm^3^ in size, the mice were randomly grouped and treated intraperitoneal (i.p.) with 50 mg/kg of Olaparib, 50 mg/kg of LY2835219, 50 mg/kg of ZC-22, or 1.5 mg/kg of cisplatin alone or in combinations or with the vehicle (PBS containing 10% DMSO and 10% 2-hydroxy-propyl-β-cyclodextrin) every 2 days. Tumor volume was measured every 3–4 days, which was calculated as Length × Width^2^/2. At the end of the study, tumor and major organ tissues were harvested by surgery. For histopathological analysis, tissue samples were washed with PBS, fixed in 4% paraformaldehyde (PFA), paraffin-embedded, and sectioned into 7 μm thickness. For Western blot analysis, the tissue samples were dissociated by a tissue-tearor in RIPA buffer containing 1×protease and phosphatase inhibitors.

### 2.10. Histology

Immunohistochemical (IHC) analyses of Ki67, γH2A.X, or p-Rb in paraffin-embedded sections were performed using the Envision Kit (Dako) as described before [24,25]. Sections were analyzed by microscopy. The percentage of positively stained cells was counted blindly by two independent individuals who were unaware of the sample identification.

To detect the safety of ZC-22 monotherapy or combination therapy, sections of mouse heart, liver, spleen, lung, and kidney tissues were stained with hematoxylin and eosin (H&E) and then analyzed by microscopy.

### 2.11. Statistical Analysis

Statistical significance was determined in unpaired t-tests using the GraphPad Prism 8.0.1. Data were expressed as the means ± SD (standard deviation) or means ± SEM (standard error of mean). *p* < 0.05 were considered significant. ns, not significant, * *p* < 0.05, ** *p* < 0.01, and *** *p* < 0.001.

## 3. Results

### 3.1. Synthesis of ZC-22 and Analysis of Its Activity

Olaparib, formerly referred to as AZD2281 or KU0059436, was the first PARPi introduced into clinical practice, which was initially approved as a maintenance therapy in platinum-sensitive relapsed ovarian cancer [26] and later approved for treatment of BRCA-mutated (HR-deficiency) breast and ovarian cancers [27,28,29]. Recently, several studies indicated that a combination of Olaparib with CDK4/6i displayed therapeutic synergy in MYC highly expressed breast and ovarian cancers with HR proficiency [22,23], which represents a new treatment paradigm in these cancers beyond HR-deficiency. However, currently, there is no single compound that could combine PARP and CDK4/6 inhibition, which prompted us to develop new dual-targeting compound to improve the efficacy of PARP and CDK4/6 inhibits in breast and ovarian cancers.

**Figure 1:**
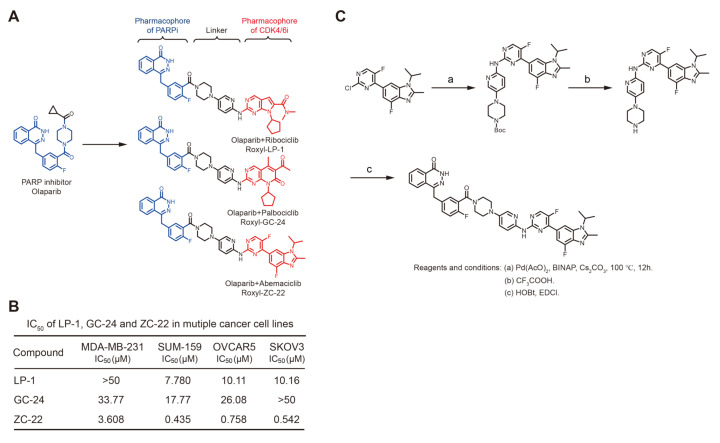
Synthesis strategy and activity assay of the novel compounds targeting both PARP and CDK4/6. (**A**) Synthesis strategy of the three novel compounds GC-24, LP-1, and ZC-22. (**B**) IC_50_ of GC-24, LP-1, and ZC-22 in breast and ovarian cancer cells. Cells were treated with increasing concentrations of GC-24, LP 1, or ZC-22 for 6 days with replenishment of medium every 2 days. Cell proliferation was analyzed by CCK-8 assays. (**C**) The reagents and conditions used for the chemical synthesis of ZC-22 in this study: (a) Pd(AcO)_2_, BINAP, CS_2_CO_3_, 1,4-Dioxane, 100 °C, 12 h; (b) CF_3_COOH, DCM, Room temperature, 6 h; (c) HOBt, EDCI, DIPEA, DMF, Room temperature, 12 h.

For this purpose, we designed a small molecule linker to integrate the pharmacophore of Olaparib and CDK4/6i, with the hope of developing a new compound that could effectively inhibit both PARP and CDK4/6. By integrating Olaparib with one of the three current CDK4/6i, Ribociclib (LEE0011), Palbociclib (PD0332991), and Abemaciclib (LY2835219), we generated three novel compounds, LP-1, GC-24, and ZC-22, respectively (Figure 1A). To analyze the anti-tumor activities of these compounds, we performed cell viability assay to calculate their IC_50_ (half maximal inhibitory concentration) value in MDA-MB-231 and SUM-159 breast cancer cells as well as OVCAR5 and SKOV3 ovarian cancer cells using the CCK-8 assay. Our data indicated that the IC_50_ values of ZC-22 were 3.608 μM, 0.435 μM, 0.758 μM, and 0.542 μM in MDA-MB-231, SUM-159, OVCAR5, and SKOV3 cells, respectively, which were much lower than the other two compounds (Figure 1B). We thus focused on ZC-22 with the highest anti-tumor activity.

We next developed an effective route to chemically synthesize highly pure ZC-22 in lab, which started from a commercially available intermediate of Abemaciclib called 6-(2-chloro-5-fluoropyrimidin-4-yl)-4-fluoro-1-isopropyl-2-methyl-1H-benzo[d]imidazole (Figure 1C). To determine the structure of this synthetic ZC-22, we further performed both ^1^H and ^13^C NMR (nuclear magnetic resonance) spectroscopy. The results confirmed the structure of ZC-22 as 4-(4-fluoro-3-(4-(6-((5-fluoro-4-(4-fluoro-1-isopropyl-2-methyl-1H-benzo[d]imidazole-6-yl)pyrimidin-2-yl)amino)pyridin-3-yl)piperazine-1-carbonyl)benzyl)phthalazin-1(2H)-one (Supplementary Figure S1A,B).

### 3.2. ZC-22 Has Betterr Anti-Tumor Efficacy Than Olaparib and Abemaciclib Alone and in Combination

**Figure 2:**
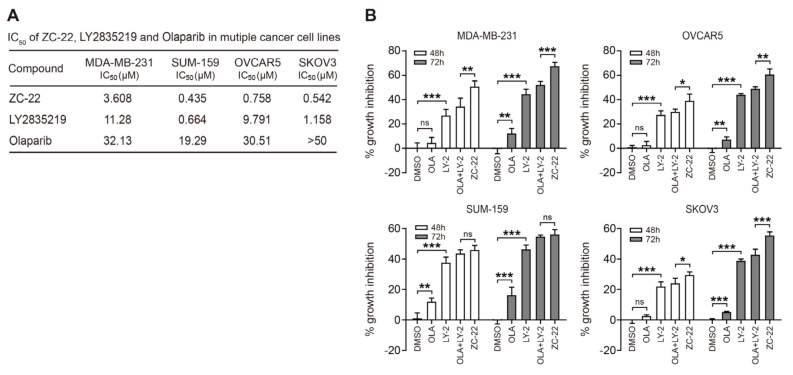
ZC-22 suppresses proliferation of breast and ovarian cancer cells. (**A**) IC_50_ of ZC-22, Olaparib, and LY2835219 (Abemaciclib) in breast and ovarian cancer cells. Cells were treated with increasing concentrations of ZC-22, Olaparib, or LY2835219 for 6 days with replenishment of medium every 2 days. Cell proliferation was analyzed by CCK-8 assays. (**B**) Cell proliferation assay of breast and ovarian cancer cells with indicated treatment. MDA-MB-231 cells were treated with 2 μM Olaparib (OLA) or LY2835219 (LY-2) alone or together (OLA + LY-2) or with ZC-22 alone for 48 or 72 h. SUM-159, OVCAR5, and SKOV3 cells were treated with 0.5 μM OLA or LY-2 alone or together or with ZC-22 alone. Cell proliferation assay was analyzed by CCK-8 assays. Values are means ± SD, *n* = 3. ns, not significant, * *p* < 0.05, ** *p* < 0.01 and *** *p* < 0.001 for comparisons with the DMSO-treated group using an unpaired *t*-test.

To investigate whether ZC-22 retained the anti-tumor efficacy of Olaparib and Abemaciclib, we compared the IC_50_ of ZC-22, Abemaciclib, and Olaparib in breast and ovarian cancer cells. Our data indicated that the anti-tumor efficacy of ZC-22 was higher than Abemaciclib and Olaparib in all of the four cancer cell lines (Figure 2A), suggesting that ZC-22 may inherit the synergistic effect of Abemaciclib and Olaparib in breast and ovarian cancer cells.

To confirm these results, we determined the growth inhibition by ZC-22 mono treatment compared with that by combined Olaparib and Abemaciclib treatment (OLA + LY-2) in breast and ovarian cancer cells. Interestingly, ZC-22 displayed a better anti-tumor efficacy than the Olaparib and Abemaciclib monotherapy, and the combination therapy in cell culture models (Figure 2B), suggesting that ZC-22 was a potential alternative for the PARPi and CDK4/6i combination therapy.

### 3.3. ZC-22 Effectively Targets CDK4/6 and PARP to Induce Cell Cycle Arrest and Apoptosis

**Figure 3:**
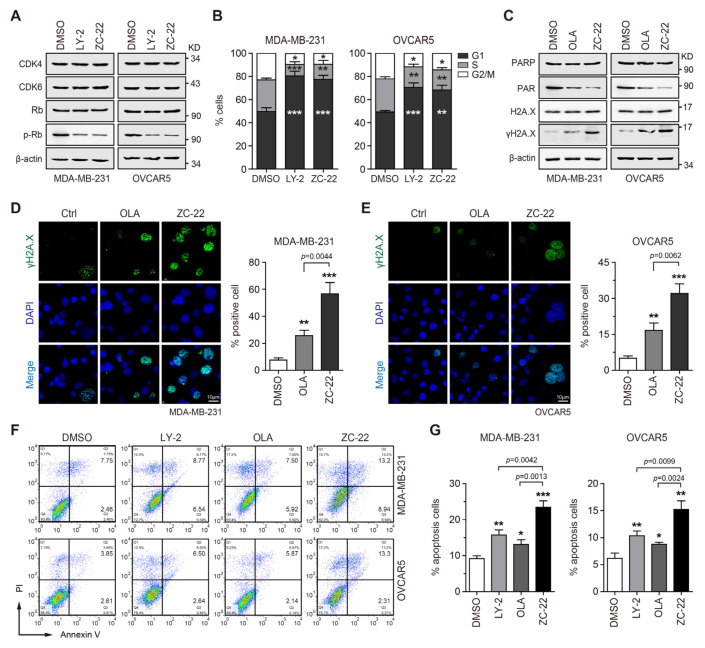
ZC-22 inhibits CDK4/6 and PARP to induce cell-cycle arrest, DNA damage, and apoptosis in breast and ovarian cancer cells. (**A**,**B**) MDA-MB-231 cells were treated with 2 μM LY2835219 (LY-2) or ZC-22. OVCAR5 cells were treated with 0.5 μM LY-2 or ZC-22, for 24 h. The levels of CDK4/6 and p-Rb/Rb were determined by Western blot (**A**). Cell-cycle distribution was analyzed by flow cytometry (**B**). Values are means ± SD, *n* = 3. * *p* < 0.05, ** *p* < 0.01, and *** *p* < 0.001 for comparisons with the DMSO-treated group at each phase in an unpaired *t*-test. (**C**) Western blot analysis of PARP, PAR, γH2A.X, and H2A.X in MDA-MB-231 cells treated with 2 μM Olaparib (OLA) or ZC-22 for 48 h (Left), or OVCAR5 cells treated with 0.5 μM OLA or ZC-22 for 48 h (Right). (**D**,**E**) Representative pictures (left) and quantification of percentage (right) of γH2A.X-positive cells in MDA-MB-231 cells treated with 2 μM OLA or ZC-22 for 24 h (**D**), or OVCAR5 cells treated with 0.5 μM OLA or ZC-22 for 24 h (**E**). Values are means ± SD, *n* = 3. * *p* < 0.05, ** *p* < 0.01, and *** *p* < 0.001 for comparisons with the DMSO-treated group. *p* value was calculated using an unpaired *t*-test. (**F**,**G**) Representative pictures (**F**) and quantification of percentage (**G**) of apoptotic cells in MDA-MB-231 cells treated with 2 μM LY-2, OLA, or ZC-22 for 48 h or in OVCAR5 cells treated with 0.5 μM LY-2, OLA or ZC-22 for 48 h. Values are means ± SD, *n* = 3. * *p* < 0.05, ** *p* < 0.01, and *** *p* < 0.001 vs. the DMSO-treated group. *p* value was calculated using an unpaired *t*-test.

CDK4/6 plays a pivotal role in the G1 to S phase cell-cycle transition through phosphorylation of retinoblastoma-associated protein (Rb) [5,6]. To investigate whether ZC-22 retained the CDK4/6 inhibitory activity of Abemaciclib, we determined the Rb phosphorylation and cell-cycle distribution in breast and ovarian cancer cells treated with Abemaciclib (LY-2) or ZC-22. The Western blot analysis indicated that ZC-22 and Abemaciclib showed equal inhibitory activity on CDK4/6-mediated phosphorylation of Rb in both MDA-MB-231 and OVCAR5 cells (Figure 3A). Similarly, the results of cell-cycle analysis demonstrated that ZC-22 dramatically arrested cell cycle at the G1/S check-point (Figure 3B and Supplementary Figure S2A–D), suggesting it was an effective CDK4/6 inhibitor.

We next analyzed the activity of ZC-22 in PARP inhibition, which leads to single-stranded break (SSB) accumulation and, consequently to double-stranded DNA breaks (DSBs) and cytotoxicity [16,17]. As expected, ZC-22 effectively inhibited PAR expression, a direct product of PARP, and subsequently induced the level of γH2A.X, a hallmark of DNA damage responses, both in MDA-MB-231 and OVCAR5 cells (Figure 3C). To confirm these results, we performed an immunofluorescence staining to detect γH2A.X foci in these cells treated with Olaparib (OLA) or ZC-22. Our data indicated that ZC-22 notably induced formation of γH2A.X foci in breast and ovarian cancer cells (Figure 3D,E). Interestingly, ZC-22 induced stronger DNA damage responses (as indicated by the level of γH2A.X and percentage of cells positive for γH2A.X foci) than Olaparib (Figure 3C–E), which was consistent with the previous studies showing that CDK4/6 inhibition impaired HR pathway [30,31,32] and consequently aggravated PARPi-induced DNA damage.

DNA damages induce cell cycle arrest to allow DNA repair, and when damages exceed the ability of cells to repair, cell death occurs, typically via apoptosis [33,34]. We thus analyzed the effect of ZC-22 on cell proliferation and apoptosis. As expected, ZC-22 strongly inhibited cell proliferation (Supplementary Figure S2E) and subsequently induced apoptosis (Figure 3F,G) both in breast and ovarian cancer cells. Taken together, these data demonstrated that ZC-22 could effectively induce cell cycle arrest and apoptosis through the inhibition of both PARP and CDK4/6 in breast and ovarian cancer cells regardless of HR status.

### 3.4. ZC-22 Monotherapy Displays Better Anti-Tumor Efficacy Than Olaparib and Abemaciclib Combination Therapy in Breast Cancer In Vivo

**Figure 4:**
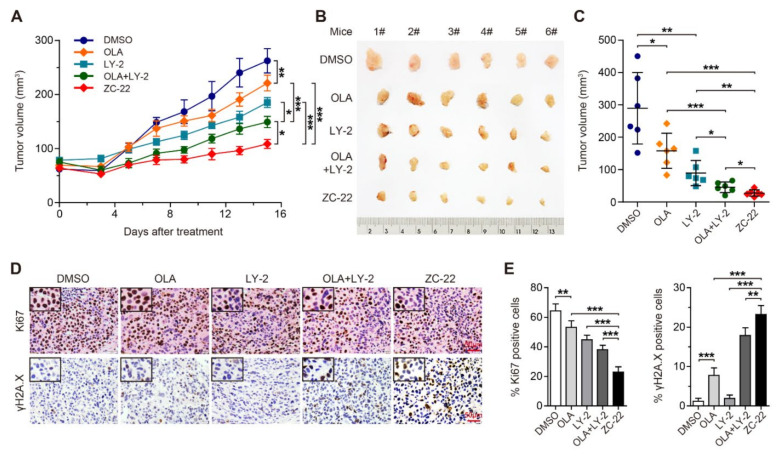
ZC-22 suppresses growth of breast cancer xenografts. Female NOD-SCID mice were injected subcutaneously with 2 × 10^6^ of MDA-MB-231 cells and treated ip daily with 50 mg/kg of Olaparib (OLA) or LY2835219 (LY-2) alone or together (OLA + LY-2) or with ZC-22 alone starting on day 10 after injection of cells when tumors were 50–80 mm^3^ in size. Tumor volumes were measured every 2–3 days. (**A**) Growth curve of the MDA-MB-231 xenograft tumors with indicated treatment. Values are means ± SEM, *n* = 6. (**B**,**C**) Pictures of (**B**) and quantification of the size of (**C**) the MDA-MB-231 xenograft tumors at the end points. Values are means ± SD, *n* = 6. (**D**) Immunohistochemical staining of Ki67 (top panels) and γH2A.X (bottom panels) in the MDA-MB-231 xenograft tumor tissues. (**E**) Quantification of Ki67 (Left) and γH2A.X (Right)-positive cells in immunohistochemical staining of the xenograft tumor tissues. Values are means ± SEM, *n* = 6. * *p* < 0.05, ** *p* < 0.01, and *** *p* < 0.001 in an unpaired *t*-test.

To further evaluate the therapeutic potential of ZC-22 in vivo, we constructed xenograft mouse models of breast cancer and then treated with Olaparib and Abemaciclib alone or together or with ZC-22. Our data indicated that the combination of Olaparib and Abemaciclib displayed better anti-tumor efficacy than Olaparib or Abemaciclib monotheray in breast cancer (Figure 4A–C). Moreover, ZC-22 showed even higher therapeutic efficacy than combined Olaparib and Abemaciclib therapy (Figure 4A–C). Similarly, immumohistochemical staining revealed that ZC-22-treated xenografts contained the lowest percentage of Ki67-positive cells and the highest percentage of γH2A.X-positive cells (Figure 4D,E), suggesting that ZC-22 could induce more severe cell-cycle arrest and DNA damages than the combination of Olaparib and Abemaciclib.

In addition, our data also indicated that ZC-22 greatly inhibited CDK4/6 and PARP signaling pathways in the xenograft tumors, as indicated by the reductions in the percentage of p-Rb-positive cells, p-Rb protein levels, and PAR protein levels (Supplementary Figure S3A–C). Data of body weight and histological analysis of the major organs in treated mice showed no obvious toxicity of the ZC-22 monotherapy (Supplementary Figure S3D,E). All together, these data demonstrated that ZC-22 monotherapy was potentially a better alternative of the Olaparib and Abemaciclib combination therapy in breast and ovarian cancer.

### 3.5. ZC-22 Sensitizes Breast and Ovarian Cancer Cells to Cisplatin In Vitro

**Figure 5:**
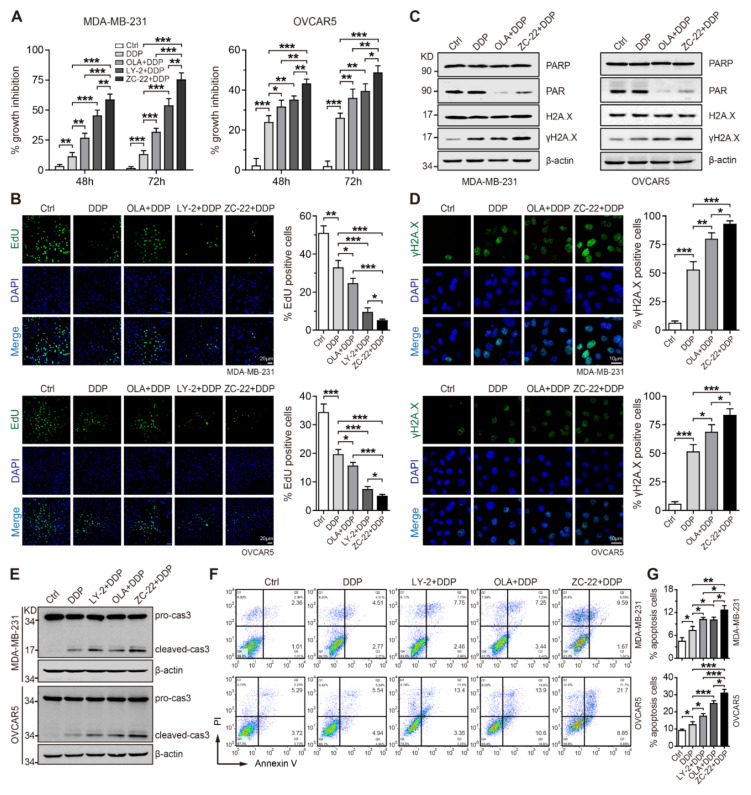
ZC-22 increases the sensitivity of breast and ovarian cancer cells to cisplatin. MDA-MB-231 or OVCAR5 cells were treated with 2.5 μM cisplatin (DDP) alone or together with 2 μM Olaparib (OLA), LY2835219 (LY-2), or ZC-22 for the indicated time. (**A**) CCK-8 assays of MDA-MB-231 (left) and OVCAR5 (right) cells treated with DDP alone or together with OLA, LY-2, or ZC-22 for 48 or 72 h, *n* = 4. (**B**) EdU incorporation analysis of MDA-MB-231 (Up) and OVCAR5 (Down) cells treated with DDP alone or together with OLA, LY-2, or ZC-22 for 24 h. Representative pictures of EdU staining are shown in the left panels, and quantification of percentage of EdU-positive cells was presented in the bar graphs on the right, *n* = 3. (**C**) Western blot analysis of PARP, PAR, and γH2A.X/H2A.X in MDA-MB-231 (left) and OVCAR5 (right) cells treated with DDP alone or together with OLA or ZC-22 for 48 h. (**D**) Representative pictures (left) and quantification of percentage (right) of γH2A.X-positive cells in MDA-MB-231 (top panels) and OVCAR5 (bottom panels) cells treated with DDP alone or together with OLA or ZC-22 for 24 h, *n* = 3. (**E**) Western blot analysis of caspase 3 (cas3) cleavage in MDA-MB-231 (top) and OVCAR5 (bottom) cells treated with DDP alone or together with LY-2, OLA, or ZC-22 for 48 h. (**F**,**G**) Representative pictures (**F**) and quantification of percentage (**G**) of apoptotic cells in MDA-MB-231 (top panels) and OVCAR5 (bottom panels) cells treated with DDP alone or together with LY-2, OLA, or ZC-22 for 48 h, *n* = 3. Values in all panels are means ± SD. * *p* < 0.05, ** *p* < 0.01, and *** *p* < 0.001 in an unpaired *t*-test.

Cisplatin and other platinum-based chemotherapeutic drugs have been extensively used for treatment of advanced breast and ovarian cancers either alone or in combination with other cytotoxic agents [35,36,37]. It is now commonly known that inhibition of DNA damage response could enhance the efficacy of cisplatin and other DNA-damaging agents [38]. As ZC-22 could notably attenuate DNA damage repair, we reason that it may improve the anti-tumor efficacy of cisplatin in breast and ovarian cancer. To test this hypothesis, we treated cells with cisplatin alone or together with Abemaciclib, Olaparib, or ZC-22. Cell viability assays indicated that ZC-22 greatly sensitized breast and ovarian cancer cells to cisplatin to a higher degree than Abemaciclib or Olaparib alone (Figure 5A and Supplementary Figure S4). Consistently, EdU incorporation assays revealed that ZC-22 significantly enhanced cisplatin-induced cell-cycle arrest in breast and ovarian cancer cells (Figure 5B).

After entering into the cells, cisplatin could react with DNA, generating monoadducts, inter- and intra-DNA strand cross-links and, subsequently, SSBs and DSBs through distortion of the double helix of DNA, which need to be repaired by the BER and HR pathways, respectively [39]. We thus investigated whether the combination of cisplatin with ZC-22 would further aggravate DNA damage in breast and ovarian cancer cells. As expected, thet combination of ZC-22 and cisplatin strongly enhanced the accumulation of γH2A.X in breast and ovarian cancer cells (Figure 5C,D), indicating that the ZC-22 and cisplatin combination could better induce DNA damage.

Since excessive DNA damage induces cell death, typically via apoptosis, we next determined apoptosis in cells treated with cisplatin alone or together with Abemaciclib, Olaparib, or ZC-22. Western blot analysis indicated that the combination of ZC-22 and cisplatin induced a stronger activation of caspase3, a key mediator of apoptosis than cisplatin alone or cisplatin together with Abemaciclib or Olaparib (Figure 5E). FACS data further confirmed that ZC-22 markedly promoted cisplatin-induced apoptosis to a higher level than Abemaciclib and Olaparib (Figure 5F,G). These findings suggest that combination of ZC-22 and cisplatin may be an effective strategy for the treatment of breast and ovarian cancer.

### 3.6. ZC-22 Greatly Improves the Response of Breast and Ovarian Cancer Cells to Cisplatin Treatment in Vivo

**Figure 6:**
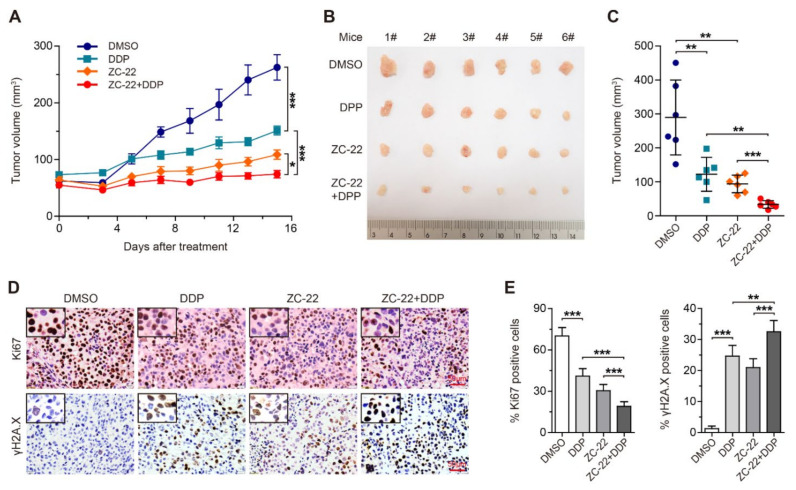
ZC-22 sensitizes breast cancer cells to cisplatin treatment in vivo. Female NOD-SCID mice were injected subcutaneously with 2 × 10^6^ of MDA-MB-231 cells and treated ip daily with 1.5 mg/kg of cisplatin (DDP) or 50 mg/kg of ZC-22 alone or together (ZC-22 + DDP) starting on day 10 after injection of cells when tumors were 50–80 mm^3^ in size. Tumor volumes were measured every 2–3 days. This experiment was simultaneously performed with that of Figure 4 and shared the same control (DMSO) group. (**A**) Growth curve of the MDA-MB-231 xenograft tumors with indicated treatment. Values are means ± SEM, *n* = 6. (**B**,**C**) Pictures (**B**) and quantification of the size (**C**) of the MDA-MB-231 xenograft tumors at the end points. Values are means ± SD, *n* = 6. (**D**) Immunohistochemical staining of Ki67 (top panels) and γH2A.X (bottom panels) in the MDA-MB-231 xenograft tumor tissues. (**E**) Quantification of Ki67 (left) and γH2A.X (right)-positive cells in immunohistochemical staining of the xenograft tumor tissues. Values are means ± SEM, *n* = 6. * *p* < 0.05, ** *p* < 0.01, and *** *p* < 0.001 in an unpaired *t*-test.

To further investigate whether the combination of ZC-22 with cisplatin has therapeutic synergy in breast and ovarian cancer, we treated breast cancer xenografts with cisplatin and ZC-22 alone or together. Our data indicated that either cisplatin or ZC-22 alone could effectively inhibit growth of the xenograft tumors, while the combination of ZC-22 and cisplatin displayed further tumor inhibition (Figure 6A–C). Moreover, a histological analysis of tumor sections revealed that the combination of ZC-22 and cisplatin dramatically suppressed the expression of Ki67 and increased the accumulation of γH2A.X (Figure 6D,E), suggesting that it was an effective strategy for the treatment of breast cancer.

**Figure 7:**
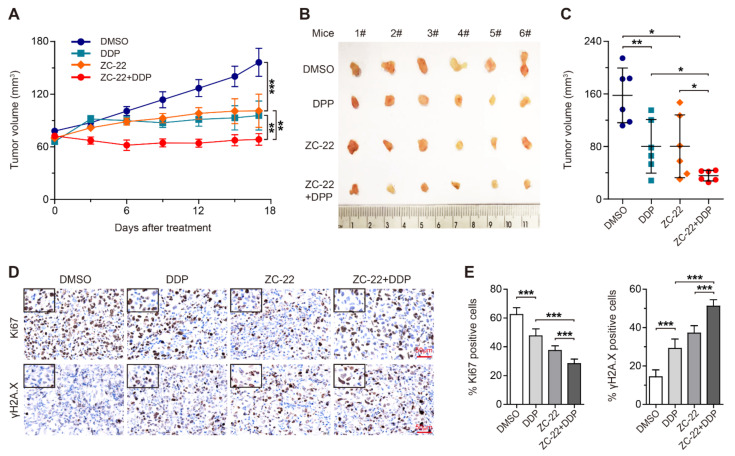
ZC-22 increases the efficacy of cisplatin treatment in ovarian cancer cells in vivo. Female NOD-SCID mice were injected subcutaneously with 8 × 10^6^ of OVCAR5 cells and treated ip daily with 1.5 mg/kg of cisplatin (DDP) or 50 mg/kg of ZC-22 alone or together (ZC-22 + DDP) starting on day 10 after injection of cells when tumors were 50–70 mm^3^ in size. Tumor volumes were measured every 2–3 days. (**A**) Growth curve of the OVCAR5 xenograft tumors with indicated treatment. Values are means ± SEM, *n* = 6. (**B**,**C**) Pictures (**B**) and quantification of the size (**C**) of the OVCAR5 xenograft tumors at the end points. Values are means ± SD, *n* = 6. (**D**,**E**) Representative pictures (**D**) and quantification of percentage (**E**) of Ki67 and γH2A.X-positive cells in immunohistochemical staining of the OVCAR5 xenograft tumor tissues. Values are means ± SEM, *n* = 6. * *p* < 0.05, ** *p* < 0.01, and *** *p* < 0.001 in an unpaired *t*-test.

We next performed the same experiment in xenograft mouse models of ovarian cancer. As expected, ZC-22 significantly sensitized ovarian cancer cells to cisplatin treatment (Figure 7A–C). Meanwhile, the combination of ZC-22 and cisplatin remarkably inhibited cell proliferation and Rb phosphorylation and aggravated DNA damage to a higher level than ZC-22 or cisplatin alone in ovarian cancer cells (Figure 7D,E and Supplementary Figure S5A,B). In addition, data of body weight and H&E staining of sections of the major organs of the mice treated with ZC-22 and cisplatin showed no obvious toxicity (Supplementary Figure S5C,D), suggesting that ZC-22 was a safe treatment for breast and ovarian cancer. Taken together, these results demonstrated that combination of ZC-22 and cisplatin may be an effective strategy in extending the use of these agents beyond HR-deficient cancers and in improving clinical outcomes of breast and ovarian cancers.

## 4. Discussion

Recently, therapeutic synergy between PARPi and CDK4/6i has been demonstrated in breast and ovarian cancer patients with HR proficiency [22,23]. Based on these findings, we developed a new compound ZC-22 that could inhibit both PARP and CDK4/6. This dual targeting compound displayed better anti-tumor efficacy than PARPi or CDK4/6i alone and in combination. Moreover, ZC-22 greatly improved the response of breast and ovarian cancer cells to cisplatin treatment.

PARPi are effective therapeutic agents that induce synthetic lethality in HR-deficient cancers, which are most commonly caused by germline or somatic mutations in BRCA1/2 genes [17,18]. In BRCA-mutated tumor cells, PARPi-induced DSBs cannot be repaired by the error-free homologous recombination (HR) pathway and instead are repaired by the alternative, error-prone non-homologous end-joining (NHEJ) pathways, which results in genome instability and consequent cytotoxicity [18,40,41,42]. Recent studies have revealed additional mechanisms underlying PARPi toxicity. It is reported that PARPi can trap PARP1/2 at the DNA damaged sites to form toxic PARP–DNA complexes, which is named “PARP trapping” [42,43]. PARP trapping in turn destabilizes replication forks and causes fork breakage, which needs to be resolved by BRCA-dependent HR repair [15,44,45]. In addition, PARP1/2 themselves are critical for Mre11-dependent replication restart at stalled replication forks, and their inhibition by PARPi also causes genome instability and synthetic lethality in HR-deficient cancer cells [42,46,47].

Hyper-activation of CDK4/6 exists in most of the breast and ovarian cancer cells, resulting in uncontrolled cell proliferation [5]. Therefore, CDK4/6i have emerged as an attractive therapeutic strategy for cancer treatment [6]. It is well known that CDK4/6 inhibition reduces proliferation and leads to cell cycle arrest and, consequently, to cell death. Previous studies reported that CDK4/6 also played a central role in DNA replication and repair pathways by regulating the RB/E2F axis [31,48]. RB, a direct substrate of CDK4/6, functions as a transcriptional repressor of the E2F family of transcription factors, which control the transcription of a set of genes required for DNA damage response and repair, especially for the HR-mediated DNA repair. Therefore, selective CDK4/6 inhibition prevents DNA repair upon treatment with DNA-damaging agents, such as anthracyclines and PARPi [31,48]. Recently, therapeutic synergy for combination of CDK4/6i and PARPi has been demonstrated in various solid tumors, including those of the breast, ovarian, and pancreas [22,23,48], which provides a promising approach for treatment of these cancers regardless HR status. In fact, current evidences indicate that inhibition of CDK6 but not CDK4 results in defective DNA repair and increased DNA damage, suggesting a role of CDK6 in controlling DNA replication and repair processes [30]. All together, these studies support our findings that combination of CDK4/6 and PARP inhibition by ZC-22 displays anti-tumor efficacy in breast and ovarian cancers beyond HR deficiency.

Unexpectedly, we found that ZC-22 showed a stronger anti-tumor effect than the combination of CDK4/6i Abemaciclib and PARPi Olaparib in breast and ovarian cancer at the same concentration (Figure 2B and Figure 5). It is likely that ZC-22 binds to CDK4/6 and PARP at the same time and causes crosslink of these two proteins, which traps a part of PARP in cytoplasm with CDK4/6 to prevent it from binding to DNA damage sites in nucleus, resulting in stronger inhibition of DNA damage repair. More studies, such as immunofluorescence staining and co-immunoprecipitation, are needed to confirm this hypothesis and to define the mechanism underlying the potent anti-tumor efficacy of ZC-22.

Clinically approved platinum-based drugs such as cisplatin and carboplatin represent valuable options for the treatment of advanced breast and ovarian cancers either alone or in combination with other drugs [36,37]. Once into the cells, cisplatin is activated when the chloride atoms are displaced by water molecules, forming an electrophile with affinity towards sulfhydryl groups on proteins and nitrogen donor atoms on nucleic acids [37]. These aquated platinum salts react with DNA, generating monoadducts, inter- (ICL) and intra-DNA strand cross-links, which distort the double helix of DNA to block both DNA replication and DNA transcription, consequently generating to SSBs and DSBs [39]. Generally, cisplatin-generated DNA bulky lesions can be efficiently repaired by the nucleotide excision repair (NER) and HR pathways. In addition, multiple studies have highlighted that base-excision repair (BER) is required for repair of platinum-induced DNA ICLs and modulation of other indirect effects generated by cisplatin exposure, although not being directly active on bulky DNA lesions [39]. Therefore, any defect in one of these DNA repair pathways, particularly HR and BER, can increase the efficacy of platinum salts, which is consistent with our findings that the inhibition of both CDK4/6 and PARP by ZC-22 greatly improves the response of breast and ovarian cancer to cisplatin treatment.

In conclusion, we developed a novel compound, ZC-22, as a CDK4/6 and PARP dual inhibitor, which displays high therapeutic potential for advanced breast and ovarian cancer patients regardless their HR status, either alone or in combination with platinum-based agents.

## Data Availability

Not applicable.

## Supplementary Materials

The following supporting information can be downloaded at: https://www.mdpi.com/article/10.3390/ijms23052892/s1.
