# Supplementary material for: A Novel CDK4/6 and PARP Dual Inhibitor ZC-22 Effectively Suppresses Tumor Growth and Improves the Response to Cisplatin Treatment in Breast and Ovarian Cancer
Source: Int J Mol Sci. 2022 Mar 7;23(5):2892. doi: 10.3390/ijms23052892 (PMC8911181; doi:10.3390/ijms23052892)
Supplement: Supplementary file 1 [file ijms-23-02892-s001.zip › ijms-1579490-supplementary.pdf]

International Journal of
Molecular Sciences

MDPI

Article

A Novel CDK4/6 and PARP Dual Inhibitor ZC-22 Effectively Suppresses Tumor Growth and Improves the Response to Cisplatin Treatment in Breast and Ovarian Cancer

Chenchen Tian [1,†], Yufan Wei [1,†], Jianjun Li [1], Zhi Huang [1], Qiong Wang [1], Yingxue Lin [1], Xingping Lv [1], Yanan Chen [1], Yan Fan [1], Peiqing Sun [2], Rong Xiang [1], Antao Chang [3,*] and Shuang Yang [1,*]

[1] Tianjin Key Laboratory of Tumor Microenvironment and Neurovascular Regulation, School of Medicine, Nankai University, Tianjin 300071, China; tianchenchen@ucas.ac.cn (C.T.); weiyufan@mail.nankai.edu.cn (Y.W.); lijianjun@mail.nankai.edu.cn (J.L.); huangzhi@mail.nankai.edu.cn (Z.H.); qiongwang2022@gmail.com (Q.W.); linyingxue@mail.nankai.edu.cn (Y.L.); lvxingping107@mail.nankai.edu.cn (X.L.); chenyanan@nankai.edu.cn (Y.C.); yanfan@nankai.edu.cn (Y.F.); rxiang@nankai.edu.cn (R.X.)

[2] Department of Cancer Biology and Comprehensive Cancer Center, Wake Forest Baptist Medical Center, Winston Salem, NC 27157, USA; psun@wakehealth.edu

[3] Department of Pancreatic Cancer, Tianjin Medical University Cancer Institute and Hospital, Tianjin 300060, China

* Correspondence: changantao@tjmuch.com or changantao@nankai.edu.cn (A.C.); yangshuang@nankai.edu.cn (S.Y.); Tel.: +86-022-2350-9557 (A.C.)

† These authors contribute equally to the work.

This file includes Figure S1 to S5.

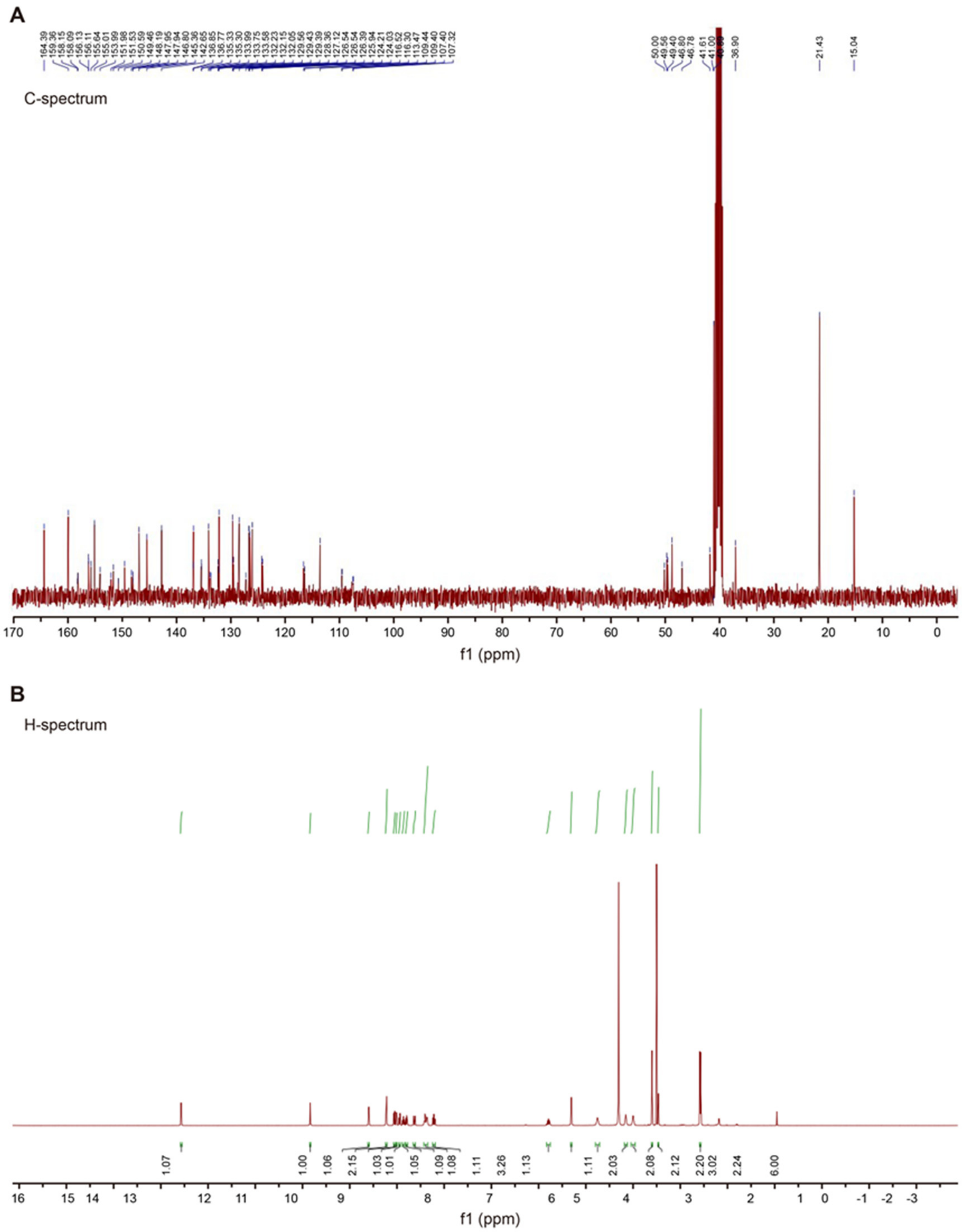

Figure S1. Chemical characterization of ZC-22. **(A)**^{13}C NMR spectrum of ZC-22. Parameters: (101 MHz, DMSO-d6) δ 164.30, 159.86, 158.08, 156.13, 155.01, 148.19, 147.94, 146.80, 145.36, 142.65, 136.77, 135.34, 133.99, 132.23, 132.05, 129.56, 129.43, 128.36, 126.54, 126.39, 125.94, 124.21, 116.52, 113.47, 109.40, 49.56, 49.40, 48.60, 46.78, 41.61, 40.89, 36.90, 21.43, 15.04. ESI-HRMS m/z calcd745.2904 [M+H]$^+$, found 745.2976[M+H]$^+$.**(B)**^{1}H NMR spectrum of ZC-22. Parameters: (400 MHz, DMSO-d6) δ 12.57 (s, 1H), 9.83 (s, 1H), 8.60 (d, J = 3.9 Hz, 1H), 8.22 (dd, J = 7.4, 1.2 Hz, 2H), 8.05 (d, J = 9.1 Hz, 1H), 8.01 (d, J = 2.9 Hz, 1H), 7.94 (dt, J = 8.2, 1.1 Hz, 1H), 7.86 (ddd, J = 8.1, 7.2, 1.5 Hz, 1H), 7.80 (dd, J = 7.8, 1.2 Hz, 1H), 7.63 (dd, J = 11.8, 1.3 Hz, 1H), 7.44 – 7.33 (m, 3H), 7.21 (t, J = 9.0 Hz, 1H), 4.90 – 4.70 (m, 1H), 4.30 (s, 2H), 3.75 (s, 2H), 3.15 (t, J = 5.2 Hz, 2H), 3.00 (s, 2H), 2.60 (s, 3H), 2.46 (p, J = 1.9 Hz, 2H), 1.57 (d, J = 6.9 Hz, 6H).

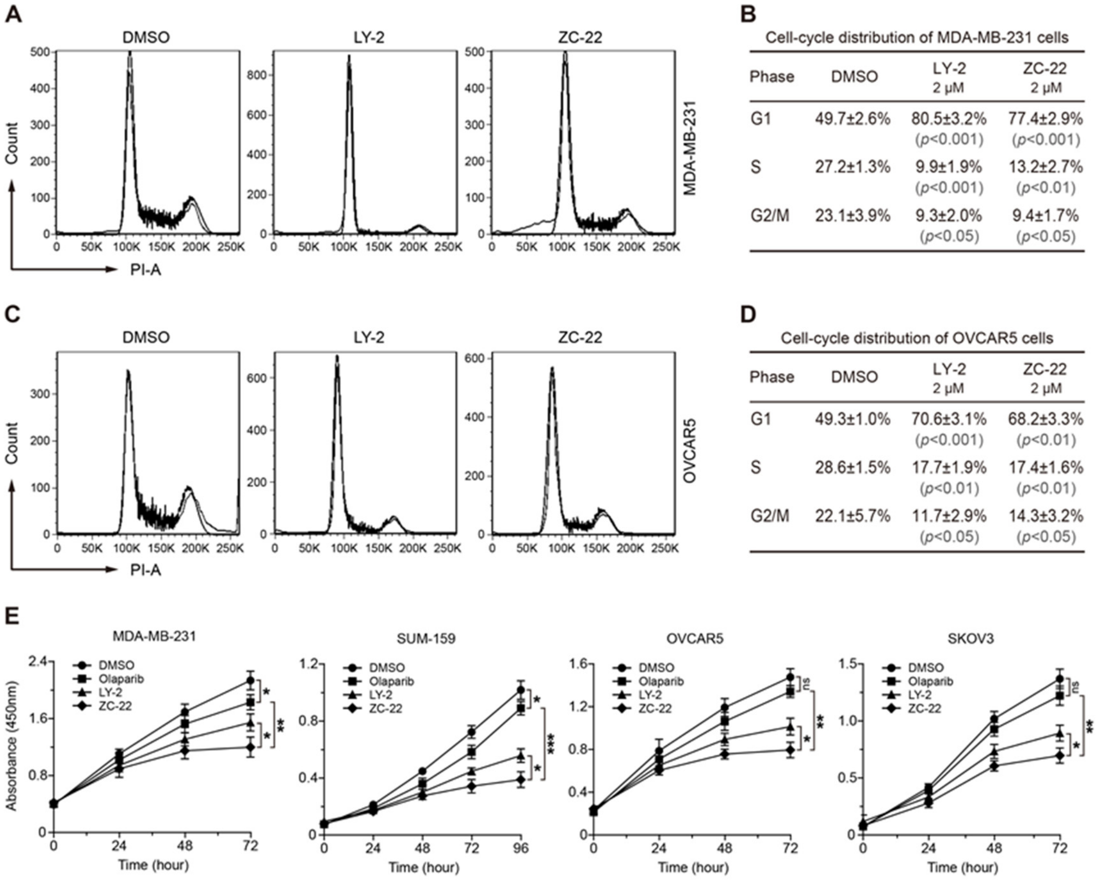

Cell-cycle distribution of MDA-MB-231 cells

Phase	DMSO	LY-2 2 μM	ZC-22 2 μM
G1	49.7±2.6%	80.5±3.2% (*p*<0.001)	77.4±2.9% (*p*<0.001)
S	27.2±1.3%	9.9±1.9% (*p*<0.001)	13.2±2.7% (*p*<0.01)
G2/M	23.1±3.9%	9.3±2.0% (*p*<0.05)	9.4±1.7% (*p*<0.05)

Cell-cycle distribution of OVCAR5 cells

Phase	DMSO	LY-2 2 μM	ZC-22 2 μM
G1	49.3±1.0%	70.6±3.1% (*p*<0.001)	68.2±3.3% (*p*<0.01)
S	28.6±1.5%	17.7±1.9% (*p*<0.01)	17.4±1.6% (*p*<0.01)
G2/M	22.1±5.7%	11.7±2.9% (*p*<0.05)	14.3±3.2% (*p*<0.05)

Figure S2. ZC-22 induces cell cycle arrest and inhibits proliferation of breast and ovarian cancer cells. **(A,B)** Cell-cycle analysis of MDA-MB-231 cells treated with 2 μM LY2835219 (LY-2) or ZC-22 for 24 h. Representative pictures of cell-cycle staining are shown in **(A)**, and quantification of percentage of cells at each phase is listed in **(B)** as means ± SD, n = 3. p value was calculated using an unpaired t-test for comparisons with the DMSO-treated group at each phase. **(C-D)** Cell-cycle analysis of OVCAR5 cells treated with 0.5 μM LY-2 or ZC-22 for 24 h. Representative pictures are shown in **(C)**, and quantification of percentage of cells at each phase is listed in **(D)** as means ± SD, n = 3. p value was calculated using an unpaired t-test for comparisons with the DMSO-treated group. **(E)** Cell proliferation assay of breast and ovarian cancer cells with indicated treatment in a time course manner. MDA-MB-231 cells were treated with 2 μM Olaparib, LY-2, or ZC-22 alone. SUM-159, OVCAR5 and SKOV3 cells were treated with 0.5 μM Olaparib, LY-2, or ZC-22 alone. Cell proliferation was analyzed by CCK8 assays at the indicated time point. Values are means ± SD, n = 3. ns, not significant, * $p < 0.05$,** $p < 0.01$, and *** $p < 0.001$ in the unpaired t-test.

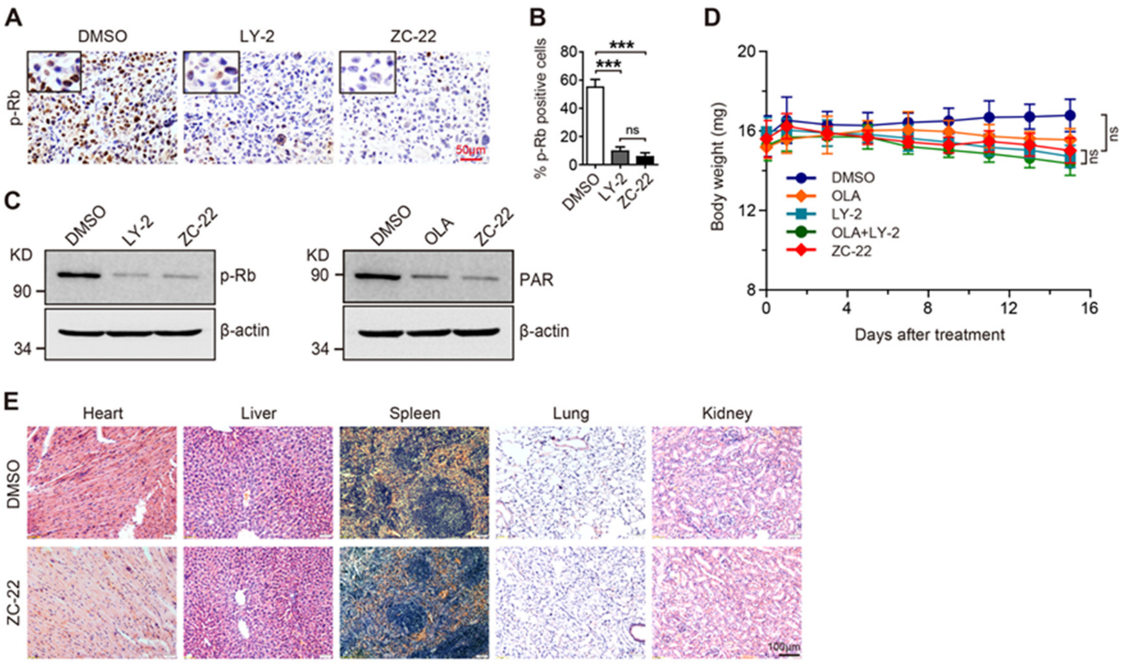

Figure S3. ZC-22 inhibits signaling of CDK4/6 and PARP in xenograft tumors of breast cancer. Female NOD-SCID mice were injected subcutaneously with 2×10⁶ of MDA-MB-231 cells and treated ip daily with 50 mg/kg of Olaparib (OLA) or LY2835219 (LY-2) alone or together (OLA+LY-2), or ZC-22 starting on day 10 after injection of cells when tumors were 50–80 mm³ in size. Representative pictures of **(A)** and quantification of percentage of **(B)** phosphorylated Rb (p-Rb)-positive cells in immunohistochemical staining of the MDA-MB-231 xenograft tumor tissues. Values are means ± SEM, n = 6. ns, not significant, and *** $p < 0.001$ in the unpaired t-test. **(C)** Western blot analysis of p-Rb (Left) and PAR (Right) in the MDA-MB-231 xenograft tumor tissues with indicated treatment. **(D)** Body weights of MDA-MB-231 tumor burden mice with indicated treatment. Values are means ± SD, n = 6. ns, not significant in unpaired t-test. **(E)** H&E staining of the heart, liver, spleen, lung, and kidney tissues of tumor-bearing mice treated with DMSO or ZC-22, showing the lack of toxicity of ZC-22 treatment.

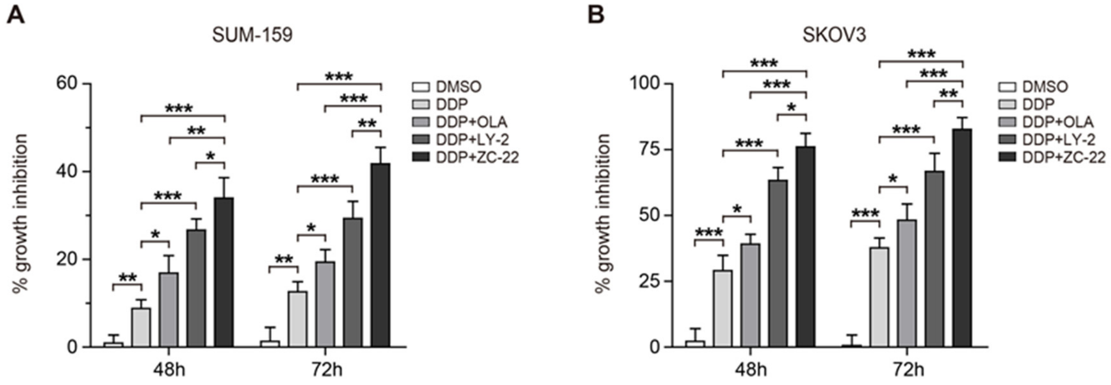

Figure S4. ZC-22 increases the sensitivity of breast and ovarian cancer cells to cisplatin. **(A)** CCK8 assay of SUM-159 cells treated with 2.5 μM cisplatin (DDP) alone or together with 0.5 μM Olaparib (OLA), LY2835219 (LY-2), or ZC-22 for 48 or 72 h. Values are means ± SD, n = 4. * $p < 0.05$, ** $p < 0.01$, and *** $p < 0.001$ using unpaired t-test. **(B)** CCK8 assay of SKOV3 cells treated with 5 μM DDP alone or together with 0.5 μM OLA, LY-2, or ZC-22 for 48 or 72 h. Values are means ± SD, n = 4. * $p < 0.05$, ** $p < 0.01$, and *** $p < 0.001$ using the unpaired t-test.

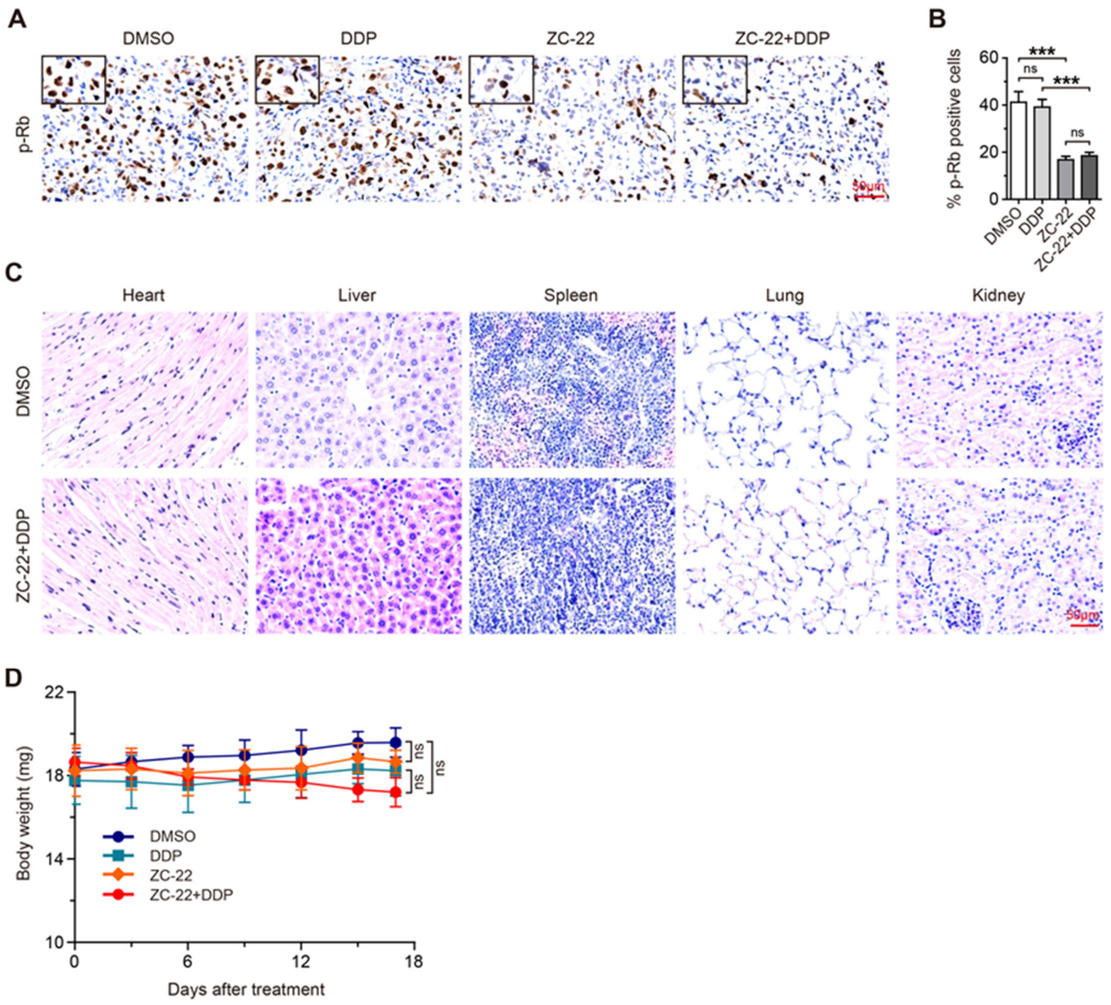

Figure S5. ZC-22 suppresses CDK4/6 signaling in xenograft tumors of ovarian cancer. Female NOD-SCID mice were injected subcutaneously with 8×10^6 of OVCAR5 cells and treated ip daily with 1.5 mg/kg of cisplatin (DDP) or 50 mg/kg of ZC-22 alone or together (ZC-22+DDP) starting on day 10 after injection of cells when tumors were 50–70 mm^3 in size. **(A)** Immunohistochemical staining of phosphorylated Rb (p-Rb) in the OVCAR5 xenograft tumor tissues. **(B)** Quantification of the p-Rb-positive cells in immunohistochemical staining of the OVCAR5 xenograft tumor tissues in **(A).** Values are means ± SEM, $n = 6$. ns, not significant, and *** $p < 0.001$ in unpaired t-test. **(C)** H&E staining of the heart, liver, spleen, lung, and kidney tissues of tumor-bearing mice treated with DMSO or ZC-22 together with DDP, showing the lack of toxicity of combined ZC-22 and DDP treatment. **(D)** Body weights of OVCAR5 tumor burden mice with indicated treatment. Values are means ± SD, $n = 6$. ns, not significant in unpaired t-test.
